# Technology-Based HIV Prevention Interventions for Men Who Have Sex With Men: Systematic Review and Meta-Analysis

**DOI:** 10.2196/63111

**Published:** 2025-04-28

**Authors:** Wenting Huang, Daniel Stegmueller, Jason J Ong, Susan Schlueter Wirtz, Kunru Ning, Yuqing Wang, Guodong Mi, Fei Yu, Chenglin Hong, Jessica M Sales, Yufen Liu, Stefan D Baral, Patrick S Sullivan, Aaron J Siegler

**Affiliations:** 1 Behavioral, Social, and Health Education Sciences Rollins School of Public Health Emory University Atlanta, GA United States; 2 Department of Political Science Duke University Durham, NC United States; 3 Melbourne Sexual Health Centre Alfred Health Melbourne Australia; 4 Central Clinical School, Faculty of Medicine Nursing and Health Sciences Monash University Melbourne Australia; 5 Department of Epidemiology Rollins School of Public Health Emory University Atlanta, GA United States; 6 Danlan Beijing Media Limited Beijing China; 7 School of Social Work University of Connecticut Hartford, CT United States; 8 National Center for AIDS/STD Control and Prevention Chinese Center for Disease Control and Prevention Beijing China; 9 Department of Epidemiology Johns Hopkins School of Public Health Baltimore, MD United States

**Keywords:** HIV, men who have sex with men, telemedicine, systematic review, Bayesian, meta-analysis

## Abstract

**Background:**

There remain unmet HIV prevention needs in China, particularly among gay, bisexual, and other men who have sex with men. Technology-based interventions are increasingly used in HIV prevention worldwide.

**Objective:**

We aimed to conduct a systematic review and meta-analysis of studies to assess the effectiveness of technology-based HIV prevention interventions to improve HIV testing and consistent condom use in China.

**Methods:**

We searched English-language (PubMed and MEDLINE, Embase, and Web of Science) and Chinese-language (Wanfang, WEIPU, and China National Knowledge Infrastructure) databases for technology-based HIV prevention intervention studies published between January 1, 2004, and September 30, 2021. Eligible studies were technology-based HIV prevention intervention studies with outcomes of HIV testing or condom use among men who have sex with men or transgender women using randomized controlled or nonrandomized pretest-posttest designs in China. The intervention technologies identified were apps, web pages, and other types of electronic communications (eg, email, SMS text messages, and video messages). A Bayesian meta-analysis was conducted to estimate the pooled effect size and 95% credible interval (CrI). We added study and intervention features as covariates to explore their associations with the study effects. Study quality was assessed using the integrated quality criteria for review of multiple study designs. Publication bias was assessed using funnel plots and robust Bayesian meta-analyses.

**Results:**

We identified 1214 and 1691 records from English-language and Chinese-language databases, respectively. A total of 141 records entered full-text screening, and 24 (17%) studies were eligible for the review. Approximately half (14/24, 58%) of the interventions were delivered through social media platforms, predominantly using message-based communication. The remaining studies used email and web-based platforms. The pooled effect sizes estimated were an absolute increase of 20% (95% CrI 10%-30%) in HIV testing uptake and an absolute increase of 15% (95% CrI 5%-26%) in consistent condom use. The pooled point estimate of the effect of randomized controlled trials was smaller than that of nonrandomized studies for HIV testing uptake (16% vs 23%) and consistent condom use (10% vs 19%), but their CrIs largely overlapped. Interventions lasting >6 months were associated with a 35% greater uptake of HIV testing (95% CrI 19%-51%) compared to those lasting 6 months.

**Conclusions:**

Technology-based HIV prevention interventions are promising strategies to improve HIV testing uptake and consistent condom use among men who have sex with men in China, with significant effects found across a broad array of studies and study designs. However, many studies in this review did not include randomized designs or a control group. More rigorous study designs, such as randomized controlled trials, are needed, with outcome measurements that address the limitation of self-report outcomes to inform the development and implementation of future intervention programs.

**Trial Registration:**

PROSPERO CRD42021270856; https://www.crd.york.ac.uk/PROSPERO/view/CRD42021270856

## Introduction

### Background

HIV remains an important health concern and an area of public health efforts in China, with a particular focus on HIV transmission among gay, bisexual, and other men who have sex with men due to high and sustained risk of HIV infection. A cross-sectional survey of 47,000 men who have sex with men from 61 cities in China in 2008 to 2009 found an overall HIV prevalence of 4.9% [1]. Since 2010, the HIV prevalence among men who have sex with men has emerged as the highest population-specific prevalence in China, increasing from 5.7% to 7.8% by 2014 according to China’s national HIV sentinel surveillance system [2]. Studies have suggested that transgender women may face an even higher HIV prevalence (>10%) than cisgender men who have sex with men [3-6].

Applying technology-based approaches to deliver HIV prevention information has been proposed as a critical part of engaging sexual minority populations that are disproportionately missed in HIV prevention and care in China [7]. These technology-based interventions include social media messaging [8], web-based HIV referral services [9], chatbots [10], and gay social networking apps [11-13]. Given that >80% of men who have sex with men do not disclose their sexual orientation to health care providers in China [14], technology-based approaches both allow for improved targeting of interventions to this hidden population and represent an opportunity to offer men who have sex with men and transgender women easier access to HIV prevention and care services while reducing fears related to stigma and discrimination. A literature review published in 2019 found that multilevel technology-based interventions in China were effective in delivering HIV prevention and care services [15]. In addition to the various modes of technology, there is a popular all-in-one smartphone app in China, WeChat, providing opportunities to develop innovative interventions. WeChat offers multiple functions, including group chat messaging, voice and video calls, gaming, wallet and payments, and location sharing, in a single platform. WeChat has billions of monthly active users in China [16]. WeChat offers developers tools to make WeChat-based mini apps that do not have to be downloaded and installed from an app store [12]. These mini apps can include different functions and features in WeChat and can deliver complex eHealth interventions such as secondary distribution of HIV self-testing kits [17].

Several systematic reviews have identified increased use of technology-based HIV interventions over the past 2 decades and have documented mixed evidence regarding the efficacy of such interventions worldwide. After reviewing studies published in 2007 to 2019, a systematic review of technology-based HIV interventions described a trend of increasing eHealth modalities. Interventions were reported to be efficacious in increasing safe sex behaviors in the short term; only a quarter of the interventions reported maintaining the behavior change over a year [18]. Another systematic review from 2015 to 2020 found more pilot or quasi-experimental study designs than efficacy randomized controlled trials (RCTs). The review found promising feasibility and acceptability outcomes among pilot studies and some efficacious outcomes among RCTs [19]. In addition to describing the types of technology used to deliver interventions and the reported behavior changes, a few recent meta-analyses have assessed the effect sizes of technology-based interventions. A meta-analysis of eHealth interventions among men who have sex with men found a small but significant effect size for all 3 behavioral outcomes over the reviewed study period: any condomless anal intercourse with nonpaying male partners (Cohen *d*=−0.21; *P*<.001), HIV testing (Cohen *d*=0.38; *P*<.001), and having multiple sex partners (Cohen *d*=−0.26; *P*=.02) [20]. Another meta-analysis reviewing studies published between 2010 and 2018 estimated that the proportion of HIV testing uptake among study participants who were exposed to technology-based interventions meant to increase testing was 1.5 times higher than that among unexposed study participants [21]. Across the aforementioned 2 reviews, a number of intervention features were associated with greater impact on HIV testing, including interventions delivered through mainstream social media, providing self-testing kits or HIV testing referral services, placing interventions on interactive platforms, involving target users, and providing longer periods of intervention exposure [20,21]. Both meta-analyses reported high heterogeneity across their reviewed studies (*I*^2^=83.3% [20] and *I*^2^=65.2% [21]). So far, all these systematic reviews have included technology-based HIV prevention interventions worldwide, but none have been restricted to China. Given the numerous technology-based interventions and the unique WeChat platform in China, there is a need for a systematic assessment to document these intervention strategies and effects.

### Objectives

We aimed to describe technology-based HIV prevention interventions among men who have sex with men and transgender women in China, document the intervention strategies, estimate intervention effects, and explore the relationships between intervention features and effect size. This review is narrower in geographic focus than previous reviews, allowing for an understanding of local intervention effects in China. In previous reviews, small sample sizes and high heterogeneity across studies were often the major concerns given the use of conventional meta-analysis. Therefore, we conducted a Bayesian meta-analysis instead of the conventional frequentist meta-analysis to improve the precision of the pooled effect size estimation and to provide the probability of the intervention effect being >0 [22-24].

## Methods

The protocol for this review was registered in PROSPERO (registration number CRD42021270856). This review was conducted following the guidance of the *Cochrane Handbook for Systematic Reviews of Interventions* and was reported following the guidance of the PRISMA (Preferred Reporting Items for Systematic Reviews and Meta-Analyses) checklist (Multimedia Appendix 1) [25].

### Eligibility Criteria

The summary of the eligibility criteria for this systematic review is presented in Textbox 1. In this review, we used a broad definition of technology to include the internet (eg, web pages, social media platforms, and smartphone apps) and other types of electronic communication (eg, SMS text messages and video messages). The inclusion criteria and search strategy were informed by the population, intervention, comparison, and outcome framework [26], including appropriate populations for the review’s goals (eg, HIV-negative men who have sex with men or transgender women residing in China, including Taiwan, Hong Kong, and Macau), use of a technology-based intervention, an eligible study type (eg, RCTs and nonrandomized designs such as 1-group pretest-posttest studies and pretest-posttest studies with a nonequivalent comparison group), eligible study outcomes (eg, HIV seroconversion, HIV testing, condom use, pre-exposure prophylaxis initiation, and pre-exposure prophylaxis awareness and willingness), and eligible data collection time frame (January 1, 2004, to September 30, 2021) and publication time frame (January 1, 2004, to September 30, 2021). Exclusion criteria were studies that exclusively used phone calls or in-person interventions, focused on persons living with HIV, or did not have quantitative HIV prevention outcomes (eg, qualitative outcomes).

**Summary of the inclusion and exclusion criteria for the systematic review of technology-based HIV prevention interventions for men who have sex with men in China (2004-2021).**

**Inclusion criteria**

- Article or study type:
  - Randomized controlled trials
  - Nonrandomized trials with a pretest and posttest design
- Study population:
  - HIV-negative men who have sex with men
  - Transgender women
- Study setting:
  - China (including Taiwan, Hong Kong, and Macau)
- Study intervention:
  - The internet (eg, web pages, social media platforms, and smartphone apps)
  - Electronic communication (eg, SMS text messages and video messages)
- Main outcomes:
  - HIV seroconversion
  - HIV testing
  - Condom use
  - Pre-exposure prophylaxis (PrEP) initiation
  - PrEP awareness and willingness
- Quality criteria:
  - Peer-reviewed journal articles
  - Conference abstracts of the International AIDS Society (IAS) Conference and the Conference on Retroviruses and Opportunistic Infections (CROI) or the Chinese National Conference of HIV/AIDS
- Language:
  - English or Chinese
- Data collection period:
  - January 1, 2004, to September 30, 2021
- Publication period:
  - January 1, 2004, to September 30, 2021

**Exclusion criteria**

- Article or study type:
  - Reviews, narratives, commentaries, and editorials
  - Qualitative studies
- Study population:
  - Other populations
- Study setting:
  - Outside China
- Study intervention:
  - Interventions that did not use any form of internet or electronic communication
- Main outcomes:
  - Studies that exclusively used phone calls or in-person interventions
- Quality criteria:
  - Studies that were not published in a peer-reviewed journal
  - Conference abstracts that were not presented in the IAS Conference, CROI, or Chinese National Conference of HIV/AIDS
- Language:
  - All other non–English or Chinese languages
- Data collection period:
  - All periods outside January 1, 2004, to September 30, 2021
- Publication period:
  - All periods outside January 1, 2004, to September 30, 2021

### Study Identification and Selection

We searched PubMed (including PubMed Central and MEDLINE), Embase, Web of Science, Wanfang, WEIPU (including the Chinese Scientific Journals Database–), the China Academic Journals Full-Text Database and China National Knowledge Infrastructure, and the Chinese Biomedical Literature Database for studies published between January 1, 2004, and September 30, 2021. Conference abstracts were searched from the online archives of the International AIDS Society Conference and the Conference on Retroviruses and Opportunistic Infections to obtain English-language publications, as well as the archives of the Chinese National Conference of HIV/AIDS for Chinese-language publications. Search terms were first developed in English and then translated into academic Chinese terminology for searching Chinese-language databases. A full description of the search strategy can be found in Multimedia Appendix 2.

Records identified from the keyword search were managed in Covidence (Veritas Health Innovation). The selection procedure followed the PRISMA guidelines. Duplicate records were checked across English- and Chinese-language literature and were removed (n=371). In total, 2 researchers screened titles and abstracts independently to exclude records that clearly did not meet the inclusion criteria or clearly met the exclusion criteria. The eligibility of the remaining records was assessed through full-text articles. During the full-text review, all the articles (n=141) were retrieved and reviewed by 2 researchers independently. Discrepancies were resolved through discussion among the 2 researchers and a senior researcher. After 115 articles were removed according to the exclusion criteria, a total of 24 (20.9%) articles (n=11, 46% articles in English; n=12, 50% articles in Chinese; and n=1, 4% abstracts in English) were included in the final review.

### Study Quality Assessment

Study quality was assessed using the integrated quality criteria for review of multiple study designs (ICROMS), a tool developed for a wide range of study designs, including RCTs, pretest-posttest studies, cohort studies, and interrupted time-series analyses [27]. The ICROMS consists of two components: (1) a scoring system for quality of the study design and (2) a decision matrix that provides mandatory criteria and minimum requirement scores for specific study designs related to the robustness of the study. The ICROMS assessment has 7 dimensions, ranging from study aims to managing bias. Each dimension has 3 to 7 specific criteria for quality assessment. This measure has been used in previous systematic reviews of eHealth interventions [18,28]. Each study was assessed by 2 researchers independently. Discrepancies were resolved through discussion between the 2 researchers.

### Data Extraction

Data extraction for each study was conducted by 2 coders (WH, SSW, KN, and YW) independently. Data extraction for studies published in Chinese was only conducted by bilingual coders (WH, KN, and YW). A standard, Microsoft Excel–based (Microsoft Corp) tool was used to extract data on publication year, language (English or Chinese), study design, sample size, technology mode, behavioral theory applied, intervention development, intervention delivery, outcome measurement (eg, the recalled period, such as in the last time, and in the previous month), and results and analyses (eg, number of consistent condom uses, number of HIV tests, effect size measurement, and reported effect size). Several intervention features were further coded for use as covariates. These features included whether the intervention was theoretically based (ie, developed based on a behavior change theory), whether the intervention had active engagement with users (eg, using an interactive dialogue box, motivational interviewing, and one-on-one counseling sessions), whether the intervention was designed to have more than a 1-time interaction, whether intervention development and delivery involved the target population, the length of the intervention (eg, <6 months, 6 months, and >6 months), and the length of study follow-up (eg, 3 months, 6 months, and 12 months). Studies that did not report these features were coded as no. Extracted data were compared, and discrepancies were discussed with a third coder until consensus was reached.

### Data Synthesis

The primary outcomes for this review were HIV testing uptake and consistent condom use. We defined HIV testing uptake as the proportion of participants who reported receiving a facility-based HIV test during the study period or who used an HIV test kit during the study period. Consistent condom use was defined as the proportion of participants with no condomless anal sex with any partners during the study period. When the articles reported multiple follow-up assessments, the longest follow-up interval was used for the outcomes of interest. Effect sizes were calculated as mean differences either between the intervention and control or comparison groups or between the pretest and posttest time points depending on the study design. The uncertainty was assessed using SEs. SEs were extracted from the studies that reported them; for studies that did not report SEs, we manually calculated them by comparing either the intervention and control groups in the case of RCTs or the pretest and posttest outcomes or the intervention and comparison groups in the case of nonrandomized studies. Therefore, 1 HIV testing uptake outcome or 1 consistent condom use outcome were obtained for each study. Studies using noninferiority designs (2/24, 8%) were excluded from the meta-analysis given the goal of the review to estimate overall effect sizes. We decided to use the absolute effect instead of the relative effect because the absolute effect is easier to interpret for clinical practice; in contrast, relative measures sometimes provide misleading estimates [29].

Given the expected heterogeneity across the studies, a Bayesian random-effects model was used to estimate the pooled effect size and 95% credible interval (CrI) for behavior changes in HIV testing uptake and consistent condom use associated with the interventions [23]. This model assumes that reported study estimates (*y*) follow a normal distribution, with the mean equal to the true study effect and the SD equal to the reported SE. The true study effects (θ) were assumed to arise from a normal distribution, with the mean equal to the overall meta-analytic estimate (μ) and study heterogeneity (τ; Multimedia Appendix 3 [27]). In a robust version of this hierarchical model, we specified the true study effects as arising from a *t*-distribution (with estimated df) to increase robustness against potential outliers. We chose vaguely informative default priors for all model parameters and conducted prior sensitivity analyses. Multimedia Appendix 4 [20] provides more details. All models were estimated using Hamiltonian Monte Carlo sampling [30]. We ran 4 Markov chains for 20,000 iterations, dropping the first 10,000 iterations as a burn-in phase. Forest plots were used to display the estimates.

To explore relevant study and intervention features that could be associated with the study effect sizes, we added the study and intervention features as covariates to the Bayesian model. Heterogeneity and potential publication bias were evaluated qualitatively through examining contour-enhanced funnel plots, which included the statistical significance of study estimates, and quantitatively through examination of the Bayes factor with robust Bayesian meta-analysis. We also conducted subgroup analyses to estimate the pooled effect sizes and 95% CrIs by RCT and nonrandomized designs. All analyses were conducted using the *brms* and *RoBMA* packages in R (version 4.3.1; R Foundation for Statistical Computing).

## Results

### Study Characteristics

**Figure 1:**
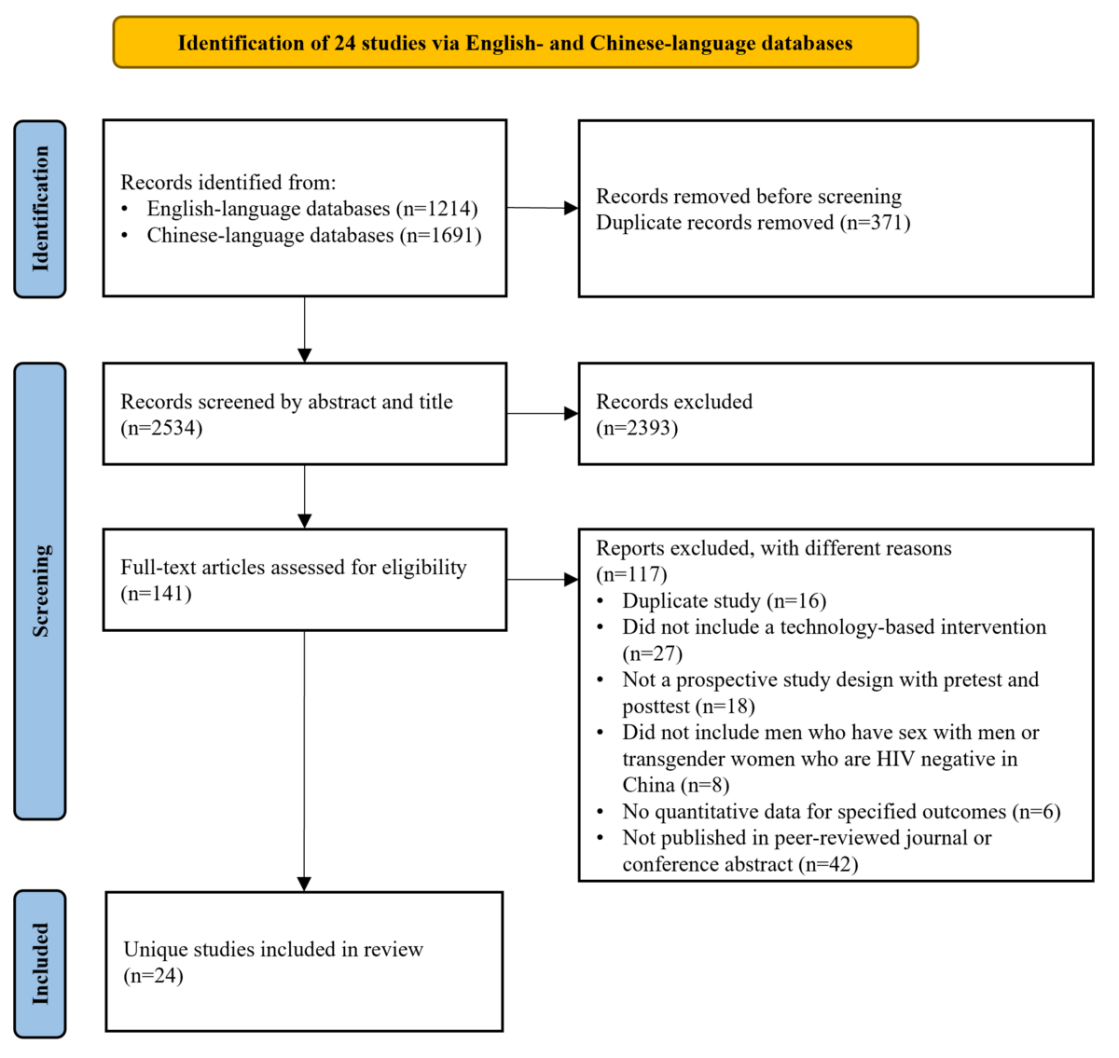
PRISMA (Preferred Reporting Items for Systematic Reviews and Meta-Analyses) flowchart of the study search and selection process.

We identified 1214 records from English-language databases and 1691 records from Chinese-language databases published between January 1, 2004, and September 30, 2021. Of these 2905 records, after removing 371 (12.77%) duplicates, 2534 (87.23%) were screened by title and abstract, and a total of 141 (4.85%) records were included in the full-text screening. After the full-text screening, 24 studies were included in the final review. The study selection process and reasons for exclusion are presented in Figure 1. Table 1 presents the characteristics of each reviewed study, including study design (with dates and sample size), technology type, intervention, theoretical framework and intervention development, intervention delivery and frequency, and reported results. Half (12/24, 50%) of the reviewed studies were published in English, and half (12/24, 50%) were published in Chinese. Most study sites (19/24, 79%) were in mainland China. Other study sites included Hong Kong (3/24, 12%) [31-33] and Taiwan (2/24, 8%) [34,35]. Although transgender women were included in our search strategy, the eligible studies were restricted to men who have sex with men. Over half (13/24, 54%) of the studies were RCTs. Of the 13 RCTs, 8 (62%) were 2-group parallel RCTs, 2 (15%) were 3-group parallel RCTs [32,36], 2 (15%) were noninferiority RCTs [37,38], and 1 (8%) was a stepped-wedge cluster RCT [39]. Among the nonrandomized studies (11/24, 46%), 9% (1/11) used a pretest-posttest design with a nonequivalent comparison group [35], 45% (5/11) used 1-group pretest-posttest designs [40-44], and 45% (5/11) used pretest-posttest designs with repeated cross-sectional surveys [45-49].

**Table 1:** Study design and intervention features in the reviewed studies in China from 2004 to 2021 (N=24).

Study and location		Study design, period, and sample size	Intervention technology	Intervention and control group	Theoretical framework and intervention development	Intervention delivery and frequency	Reported results^a^
**Traditional RCTs^b^ with 1 intervention group and 1 control group (n=8; n=6 in English and n=2 in Chinese)**							
	Cheng et al [50], 2019; mainland China	Nonblind RCT; September 2010-June 2011; N=1100	Web pages	Intervention: online interactive scenarios about HIV-related risk behaviors and health messages; control: standard HIV referral service only	TPB^c^; developed via mixed methods formative research	A total of 4 weeks—1-time delivery of 5 scenarios via web-based interactive dialogue box and weekly health messages sent via email for 3 weeks	Intervention vs control, BL^d^ to 6-month FU^e^—condomless anal sex in the previous 3 months decreased (difference in proportion=9.3%, 95% CI 1.1%-17.5%)
	Chiou et al [34], 2020; Taiwan	RCT; August 2015-May 2017; N=300	App—developed by the research team for men who have sex with men	Intervention: use of a multifunction SBS^f^ app for 6 months; control: no intervention	IMB^g^ model; developed through a qualitative study soliciting men who have sex with men’s needs, men who have sex with men user reviews, and expert review on content validity	A total of 24 weeks—a message reminding the user of the app’s functions was sent every 2 weeks, and a quiz and 2 activities related to HIV testing, safe sex, and recreational drug use were sent every 3 weeks	Intervention vs control, BL to 6-month FU—HIV and syphilis testing frequency increased (mean difference=0.217; SE 0.08); percentage of condom use during anal sex in the previous 3 months increased (difference=20.7%; SE 0.06); other outcomes: disease and safe behavior knowledge increased (difference=2.13; SE 0.21)
	Lau et al [31], 2008; Hong Kong	RCT; study dates not specified; N=477	Email	Intervention: health messages in the form of graphics, HIV-related risk behavior electronic monthly form with tailored feedback, and peer counselors; control: no intervention	Did not report that any theory was used; developed by a panel consisting of the authors	A total of 6 months—a health message was sent via email every 2 weeks and an e-form with feedback (“report card”) was sent every month	Intervention vs control, BL to 6-month FU—no change in HIV testing in the previous 6 months; no change in condom use during anal sex in the previous 6 months; other outcomes: no change in HIV- or STD^h^-related knowledge and perceptions
	Wang et al [33], 2018; Hong Kong	Nonblind RCT; study dates not specified; N=430	App—a live chat app	Intervention: an online video promoting HIV testing, another online video and an MI^i^ promoting HIVST^j^ with online real-time instructions and pretest and posttest counseling (HIVST-OIC^k^), and a free HIVST kit; control: an online video promoting HIV testing	HBM^l^; developed through literature review and a stakeholder panel discussion	1-time—online HIV testing promotion video (3 minutes), online HIVST-OIC promotion video (4 minutes), and a brief MI (15 minutes)	Intervention vs control, BL to 6-month FU—HIV testing rate increased (RR^m^=1.77, 95% CI 1.54-2.03; ARR^n^=39.1%, 95% CI 31.3%-46.9%)
	Yun et al [13], 2021; mainland China	Double-blind RCT; October 2017-June 2018; N=192	App—WeChat	Intervention: a comprehensive intervention package including 4 components—HIV risk assessment, HIV testing facility recommendation, free condoms and HIVST kit, and health education website; control: a link to an HIV health education web page	AIDS Risk Reduction Model; did not report the process of intervention development	A total of 3 months—information related to the intervention was sent every 4 weeks	Intervention vs control, BL to 3-month FU—no change in HIV testing in the previous 3 months; condom use among casual partners in the previous 3 months increased (OR^o^ 2.81, 95% CI 1.23-6.39)
	Zhu et al [12], 2019; Hefei	RCT; September 2017-June 2018; N=100	App—WeChat	Intervention: WeTest program plus watching a brief video demonstrating the use of the HIVST kit and results interpretation and 2 oral HIVST kits with standard information about regular HIV testing; control: only the demonstration video and 2 oral HIVST kits with standard information about regular HIV testing	IMB model; developed through formative in-depth interviews for intervention content, cognitive interviews for refining content, and a pilot test of the beta version of the WeTest program	A total of 6 months—2 weekly health messages about personal stories, data on HIV and STI^p^ infections among men who have sex with men, national policies, and general health concern stories of men who have sex with men and 1-time instructions for oral HIVST kit use	Intervention vs control, BL to 6-month FU—HIV testing in the previous 6 months increased (adjusted RR=1.99, 95% CI 1.03-3.84); oral HIVST in the previous 6 months increased (adjusted RR=2.17, 95% CI 1.08-4.37); no change in consistent condom use in the previous 6 months
	Li et al [51], 2020; mainland China	RCT; March 2017-April 2019; N=600	App—WeChat	Intervention: WeChat-based counseling, HIV testing, and test result notification, as well as HIV infection risk evaluation and personal behavior change suggestions; control: on-site intervention, including regular distribution of HIV education materials, in-person class, counseling, and HIV testing	Did not report that any theory was used; did not report the process of intervention development	Did not report the length of the intervention—the HIV infection risk evaluation was sent every week	Intervention vs control, BL to 6-month FU—condomless anal sex in the previous 6 months decreased (from 42% to 16% in the intervention group and from 41% to 31% in the control group; χ^2^_1_=18.0, *P*<.05)
	Xiao et al [52], 2020; Shanghai	Double-blind RCT; July 2017-June 2018; N=200	App—WeChat	Intervention: men who have sex with men–focused HIV prevention message plus regular HIV prevention message; control: regular HIV prevention message	Did not report that any theory was used; did not report the process of intervention development	A total of 12 months—a weekly regular HIV prevention message and a weekly men who have sex with men–focused HIV prevention message	Intervention vs control at the 12-month FU—no change in consistent condom use in the previous 6 months; other outcomes: knowledge increased in several AIDS knowledge items
**RCTs with 2 intervention groups and nontraditional RCTs (n=5; all in English)**							
	Lau et al [32], 2016; Hong Kong	Nonblind 3-group parallel RCT; study dates not specified; N=402	Email and web page	Intervention group 1: STD-related cognitive approach—a video about STD prevention (video 1) and a video about UAI^q^ prevention among men who have sex with men (video 2); intervention group 2: STD-related cognitive plus fear appeal imagery approach—an additional movie with fear appeal and visual imagery (video 3) plus video 1 and video 2; control: HIV-related information–based approach—received factual HIV-related text information but no video	Parallel response model; developed by an interdisciplinary panel with meetings with men who have sex with men	1-time videos—video 1 and video 2: 5 minutes; video 3: 10 minutes	Intervention group 1 vs intervention group 2, intervention group 1 vs control, and intervention group 2 vs control at the 3-month FU—no change in UAI in the previous 3 months after the intervention across the 3 groups; other outcomes: no significant association between immediate fear-related emotional responses and UAI
	Luo et al [36], 2021; Beijing, China	3-group parallel RCT; October 2017-September 2018; N=9280	App—Blued	Intervention group 1: HIV risk assessment and tailored feedback plus routine HIV education; intervention group 2: HIV risk assessment plus routine HIV education; control: routine HIV education	Did not report that any theory was used; did not report the process of intervention development	Did not report the length of the intervention and delivery frequency	Intervention group 1 vs control, BL to 12-month FU—mean number of HIV tests in the previous 12 months increased (IRR^r^=1.32, 95% CI 1.09-4.58); intervention group 1 vs intervention group 2, intervention group 1 vs control, and intervention group 2 vs control, BL to 12-month FU—no statistically significant differences in the proportion of UAI among the 3 groups
	Tang et al [37], 2016; mainland China	Noninferiority RCT; study dates not specified; N=721	Video message	Intervention group 1: a crowdsourced video promoting HIV testing; intervention group 2: a health marketing video promoting HIV testing; control: no control or comparison group	Did not report that any theory was used; the 1-minute crowdsourced video was developed via a crowdsourcing contest; the health marketing video was developed by a marketing company with public health professional guidance	1-time videos—both videos were 1 minute long	Intervention group 1 vs intervention group 2 at the 3-week FU—no difference in first-time HIV testing, with a noninferiority margin of −3%
	Tang et al [38], 2019; mainland China	Noninferiority single-blind RCT; November 2015-February 2016; N=1173	Video message	Intervention group 1: a crowdsourced video promoting condom use; intervention group 2: a social marketing video promoting condom use	Did not report that any theory was used; the 1-minute crowdsourced video was developed via a crowdsourcing contest; the social marketing video was shot by a marketing company following a script by social marketing experts and approved by young men who have sex with men	1-time—both videos were 1 minute long	Intervention group 1 vs intervention group 2 at the 3-week FU—no difference in proportion of condomless sex, with a noninferiority margin of +10%, and no difference in HIV testing in the previous 3 weeks, with a noninferiority margin of +10%; intervention group 1 vs intervention group 2 at the 3-month FU—no difference in proportion of condomless sex, with a noninferiority margin of +10%
	Tang et al [39], 2018; Guangdong and Shandong	Stepped-wedge cluster RCT; July 2016-August 2017; N=1381	App—WeChat and other social media platforms	Intervention: routine CDC^s^ and CBO^t^ promotional efforts, HIV testing promotional image, a free HIVST kit, and a local CBO-led contest for HIV testing stories; control: routine CDC and CBO promotional efforts	Did not report that any theory was used; developed through a nationwide open contest, a regional strategy designathon contests, and local participatory contests	A total of 3 months—6 HIV testing promotional images were sent via WeChat biweekly, 1-time access to a free HIVST kit, and 1-time local CBO-led contest	Intervention vs historical control, BL to 12-month FU—the proportion of HIV testing increased (difference in proportion=8.9%, 95% CI 2.2%-15.5%); the proportion of HIVST increased (RR=1.89%, 95% CI 1.5%-2.38%); no change in facility-based HIV testing; no change in condom use; no change in syphilis testing or anticipated HIV stigma
**Nonrandomized pretest-posttest designs (n=11; n=1 in English and n=10 in Chinese)**							
	Ko et al [35], 2013; Taiwan	Pretest-posttest design with nonequivalent groups using a repeated cross-sectional survey; October 2010-November 2011; n=1008 at BL and n=1037 at posttest	Web page—Facebook	Intervention: trained iPOLs^u^ shared and exchanged news, video clips, reports, and opinions and connect with others for advice and support on an online iPOL platform built on Facebook using the Web 2.0 two-way communication format; control: another nonequivalent control website with no intervention	The DOI^v^ theory; did not report the process of intervention development	A total of 6 months—no regular schedule; frequent sharing of information and 2-way conversations; a total of 432 posts, 503 comments, and 804 likes on the iPOL platform; and an estimated 959,088 people viewed the posts on the iPOL platform	Intervention vs control, BL to 6-month FU—HIV tests in the previous 6 months increased (43.89% vs 22.31%; χ^2^_1_=54.8, *P*<.01); condom use during anal sex with online sex partners increased (34.15% vs 26.19%; χ^2^_1_=13.4, *P*<.01)
	Liu et al [45], 2012; mainland China	Pretest-posttest design using a repeated cross-sectional survey; study dates not specified; n=1293 at BL and n=1014 at posttest	Web page and email	Intervention: HIV prevention messages about risk of HIV infection, safe sex practice, and HIV tests through a web page and via email; control: no control or comparison group	Did not report that any theory was used; did not report the process of intervention development	A total of 2 months—3 sessions of HIV prevention education messages; did not report the intervention frequency	BL to 3-month FU—the proportion of lifetime HIV testing increased (49% to 54%; *P*<.05); the proportion of last-time condom use in sex with men increased (65% vs 71%; *P*<.05); other outcomes: knowledge increased in several AIDS knowledge items
	Xie et al [49], 2018; Dongguan	Pretest-posttest design using a repeated cross-sectional survey; study dates not specified; n=1510 at BL and n=1321 at posttest	App—ZANK	Intervention: HIV prevention intervention (did not report any intervention details); control: no control or comparison group	Did not report that any theory was used; did not report the process of intervention development	A total of 2 months—did not report the intervention delivery and frequency	BL to 3-month FU—the proportion of lifetime HIV testing increased (49% vs 55%; χ^2^_1_=7.7, *P*<.05); consistent condom use in anal sex with men in the previous 6 months increased (50% vs 55%; χ^2^_1_=9.8, *P*<.05); the proportion of last-time condom use in anal sex with men increased (67% vs 73%; χ^2^_1_=10.8, *P*<.05); other outcomes: knowledge increased in several AIDS knowledge items
	Liu et al [40], 2014; Jining	1-group pretest-posttest design; July 2012-December 2012; N=213	App—QQ and WeChat	Intervention: one-on-one HIV prevention intervention session with CBO volunteers via QQ or WeChat and setup of a hotline for HIV and HIV testing counseling; control: no control or comparison group	Did not report that any theory was used; did not report the process of intervention development	A total of 6 months—each monthly intervention session lasted 30 minutes	BL to 6-month FU—the proportion of lifetime HIV testing increased (62% vs 76%; χ^2^_1_=9.5, *P*<.05); consistent condom use in anal sex with men in the previous 6 months increased (47% vs 61%; χ^2^_1_=7.3, *P*<.05); the proportion of last-time condom use in anal sex with men increased (56% vs 68%; χ^2^_1_=5.6, *P*<.05)
	Song et al [41], 2017; Guangxi	1-group pretest-posttest design; September 2014-December 2014; N=212	SMS text messages	Intervention: SMS text messages with HIV knowledge, condom knowledge, and HIV testing information; control: no control or comparison group	Did not report that any theory was used; did not report the process of intervention development	A total of 6 months—2 messages every week	BL to 6-month FU—no change in the proportion of HIV testing in the previous 3 months; consistent condom use in anal sex with men in the previous 3 months increased (27% vs 57%; χ^2^_1_=30.4, *P*<.05); the proportion of last-time condom use in anal sex with men increased (38% vs 73%; χ^2^_1_=3.6, *P*<.05)
	Zhang et al [44], 2014; Shandong	1-group pretest-posttest design; August 2013-May 2014; N=468	App—QQ	Intervention: HIV prevention intervention on HIV knowledge, risk of infection, condom use and safe sex practice, and HIV testing; control: no control or comparison group	Did not report that any theory was used; did not report the process of intervention development	A total of 6 months—did not report the intervention delivery and frequency	BL to 6-month FU—the proportion of lifetime HIV testing increased (30% vs 51%; df=1, *P*<.01); consistent condom use in anal sex with men in the previous 6 months increased (45% to 60%; df=1, *P*<.01); other outcomes: HIV knowledge increased (88% vs 94%; df=1, *P*<.01)
	Yan et al [43], 2013; Heilongjiang	1-group pretest-posttest design; October 2011-December 2012; N=400	App—QQ	Intervention: CBO volunteer one-on-one HIV prevention intervention session via QQ; control: no control or comparison group	Did not report that any theory was used; did not report the process of intervention development	A total of 6 months—did not report the intervention delivery and frequency	BL to 6-month FU—the proportion of lifetime HIV testing increased (57% vs 68%; df=1, *P*<.01); consistent condom use in anal sex with men in the previous 3 months increased (45% vs 60%; df=1, *P*<.01); the proportion of last-time condom use in anal sex with men increased (66% vs 82%; df=1, *P*<.05)
	Tao et al [42], 2020; Shaoxing	1-group pretest-posttest design; April 2018-June 2018; N=209	App—WeChat	Intervention: health messages delivered by a CBO and a free oral fluid–based HIVST kit; control: no control or comparison group	Did not report that any theory was used; did not report the process of intervention development	A total of 6 months—health messages were delivered biweekly	BL to 6-month FU—consistent condom use in anal sex with regular partners in the previous 6 months increased (52% to 64%; χ^2^_1_=4.4, *P*<.05); consistent condom use in anal sex with casual partners met online in the previous 6 months increased (67% to 85%; χ^2^=8.5, *P*<.05); consistent condom use in anal sex with casual partners met in person in the previous 6 months increased (58% to 89%; χ^2^_1_=5.6, *P*<.05); other outcomes: knowledge increased in several AIDS knowledge items
	Wang et al [47], 2014; Chengdu	Pretest-posttest design using a repeated cross-sectional survey; October 2012-September 2013; n=370 at BL and n=236 at posttest	Web page and app	Intervention: multidimensional intervention model including online and venue-based interventions, as well as trained peer educators conducting online counseling and introducing men who have sex with men from online to in-person venues to receive condoms, lubricant, and HIV testing; control: no control or comparison group	Did not report that any theory was used; did not report the process of intervention development	A total of 12 months—did not report the intervention delivery frequency	BL to 12-month FU—consistent condom use in anal sex in the previous 6 months increased (46% to 64%; df=1, *P*<.01)
	Wang et al [46], 2011; Yingtan	Pretest-posttest design using a repeated cross-sectional survey; August 2009-August 2010; n=135 at BL and n=134 at posttest	App—QQ	Intervention: peer education through QQ groups and on-site counseling; control: no control or comparison group	Did not report that any theory was used; did not report the process of intervention development	A total of 12 months—did not report the intervention delivery frequency	BL to 12-month FU—the proportion of lifetime HIV testing increased (10% vs 75%; χ^2^_1_=48.4, *P*<.01); consistent condom use in anal sex with men in the previous 6 months increased (24% vs 78%; χ^2^_1_=78.2, *P*<.01); the proportion of last-time condom use in anal sex with men increased (50% vs 90%; χ^2^_1_=35.8, *P*<.01); other outcomes: knowledge increased in several AIDS knowledge items
	Wang et al [48], 2009; Mianyang	Pretest-posttest design using a repeated cross-sectional survey; December 2006-January 2008; n=201 at BL and n=203 at posttest	Unclear	Intervention: POL^w^-delivered online and offline peer education; control: no control or comparison group	Did not report that any theory was used; did not report the process of intervention development	A total of 12 months—did not report the intervention delivery frequency	BL to 12-month FU—HIV testing in the previous 6 months increased (13% vs 54%; χ^2^_1_=76.5, *P*<.01)

^a^Results reported by the study authors. Point estimates for nonsignificant outcomes are left out.

^b^RCT: randomized controlled trial.

^c^TPB: theory of planned behavior.

^d^BL: baseline.

^e^FU: follow-up.

^f^SBS: safe behavior and screening.

^g^IMB: information–motivation–behavioral skills.

^h^STD: sexually transmitted disease.

^i^MI: motivational interview.

^j^HIVST: HIV self-testing.

^k^HIVST-OIC: HIVST with online real-time instructions and pretest-posttest counseling.

^l^HBM: health belief model.

^m^RR: relative risk.

^n^ARR: absolute risk reduction.

^o^OR: odds ratio.

^p^STI: sexually transmitted infection.

^q^UAI: unprotected anal intercourse.

^r^IRR: incident rate ratio.

^s^CDC: Center for Disease Control and Prevention.

^t^CBO: community-based organization.

^u^iPOL: internet popular opinion leader.

^v^DOI: diffusion of innovations.

^w^POL: popular opinion leader.

### Intervention Features

The development of approximately one-quarter of the interventions (7/24, 29%) was informed by a behavioral theory [12,13,32-35,50]. All studies guided by behavioral theories were published in English. The theories used included conventional behavioral theories such as the health belief model [33], the theory of planned behavior [50], the diffusion of innovations theory, the information–motivation–behavioral skills model [12,34], and the parallel response model [32], as well as an HIV-specific model—the AIDS Risk Reduction Model [13]. Regarding intervention content, over half (15/24, 62%) of the studies used a comprehensive intervention package rather than a single intervention [12,13,31,33-36,39,40,46-48,50-52]. Most of these comprehensive interventions (14/15, 93%) included back-and-forth interaction with participants, such as screening for HIV-related risk behaviors, peer counseling, motivational interviewing, community-based organization–led activities, and online counseling with referrals for in-person HIV tests [12,13,31,33-36,39,40,46-48,50,51]. Most of these interactions (12/15, 80%) were live conversations with a research team member or a trained volunteer [12,31,33-35,39,40,46-48,50,51]. A few studies (3/15, 20%) used automatic-reply text-based messages [13,36,50].

Over half (14/24, 58%) of the studies delivered intervention content through social media platforms and direct message functionality, half (7/14, 50%) of which were WeChat based [12,13,39,40,42,51,52]. A quarter of the studies (7/24, 29%) used SMS text messages, video messages, or email to deliver intervention content [12,31,32,37,38,41,45]. The interventions in 21% (5/24) of the studies were delivered through web pages [13,32,45,47,50]. In total, 25% (6/24) of the studies used 2 types of technologies in their interventions [12,13,31,32,45,47]. Three-quarters of studies (18/24, 75%) delivered their interventions multiple times; a third of those (6/18, 33%) did not report the frequency of intervention delivery. Among studies reporting their intervention delivery frequency, weekly (5/12, 42%) [12,35,41,50,52] and biweekly (4/12, 33%) [31,34,39,42] were the most common frequencies. The length of the interventions varied from a single time to a year, and the most frequent intervention length was 6 months (9/24, 38%) [12,31,34,35,40-44]. In terms of community engagement, less than half (10/24, 42%) of the studies reported involving men who have sex with men in intervention development [12,32-35,37-40,50], and a third of the studies (9/24, 38%) reported engaging the men who have sex with men community in intervention delivery [31,35,39,40,42,43,46-48]. Only 12% (3/24) of the studies reported engaging the men who have sex with men community in both the intervention development and delivery process [12,35,50].

### Study Quality

On the basis of the quality assessment using the ICROMS global quality scores, over two-thirds of the studies (17/24, 71%) met the score requirement (Multimedia Appendix 5). When considering the mandatory criteria, only 25% (6/24) of the studies met them, all of which were RCTs. On the basis of the quality review by study design, in the case of RCTs, 8% (1/13) of the studies did not meet the score requirement [36], and 46% (6/13) did not meet the mandatory criteria of managing bias between groups with random allocation [12,31,33,34,51,52]. The controlled pretest-posttest design study did not meet either the score requirement or the mandatory criteria [35]. Regarding uncontrolled pretest-posttest design studies (10/24, 42%), half (5/10, 50%) did not meet the minimum score requirement [40,42,45,47,48], and none of them met the mandatory criteria of mitigating the effect of no control group.

### Publication Bias

**Figure 2:**
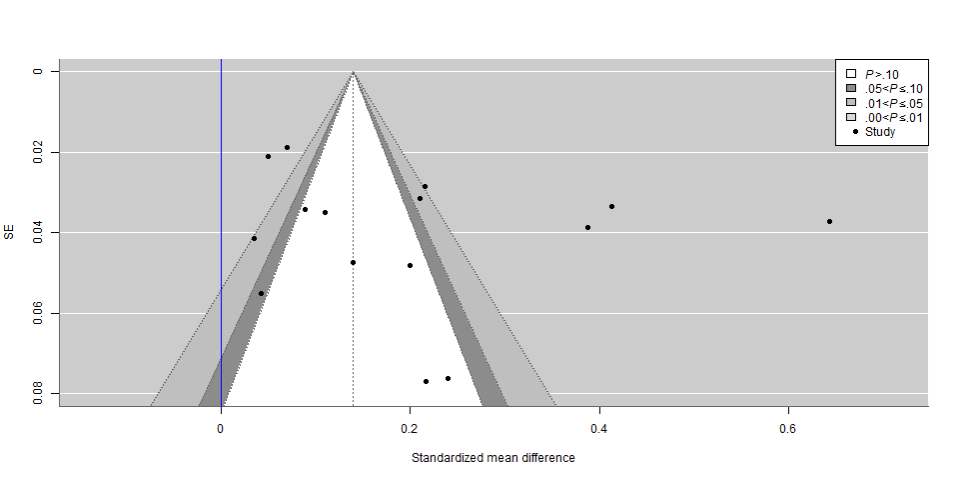
Contour-enhanced funnel plot for correlation between probability of publication and magnitude of effect for HIV testing uptake in China (2004-2021).

**Figure 3:**
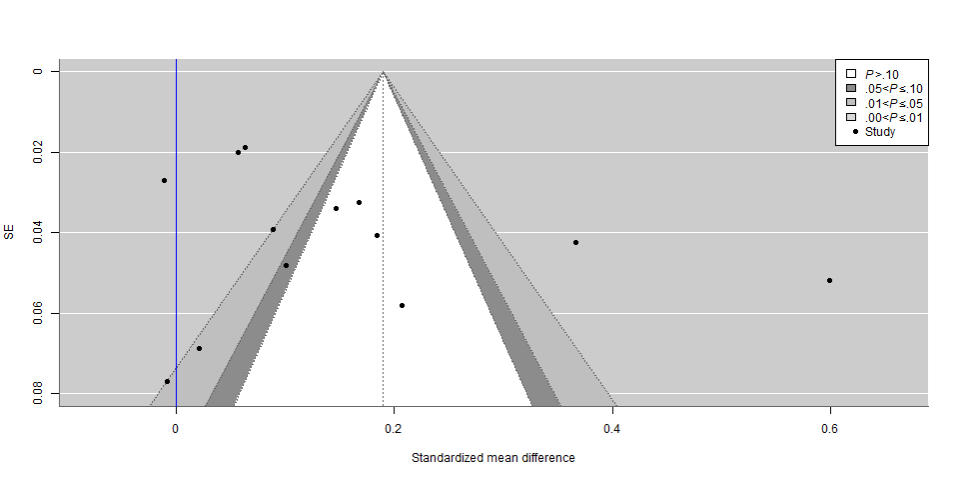
Contour-enhanced funnel plot for correlation between probability of publication and magnitude of effect for consistent condom use in China (2004-2021).

The contour-enhanced funnel plots in Figures 2 and 3 reveal an asymmetrical distribution of studies for both HIV testing uptake and consistent condom use, indicating possible publication bias. In particular, 12% (3/24) of the studies [33,46,48] and 8% (2/24) of the studies [41,46] seemed to report much larger effect sizes compared to the rest of the studies for HIV testing uptake and consistent condom use, respectively. The asymmetry regarding nonsignificant results suggests that the asymmetrical distribution was probably caused by publication bias based on statistical significance. The robust Bayesian meta-analysis results document evidence of publication bias, with a Bayes factor of 21.04 and 4.37 indicating that the likelihood of publication bias was 21.04 and 4.37 times the likelihood of no publication bias for HIV testing uptake and consistent condom use, respectively. The Egger test indicated no small-study effects as the results were not statistically significant, with a *z* statistic of 0.24 and a *P* value of .81 for HIV testing uptake and a *z* statistic of 0.37 and a *P* value of .71 for consistent condom use.

### Bayesian Meta-Analysis

For the meta-analysis, 62% (15/24) of the studies were included for the HIV testing uptake outcome, and 54% (13/24) of the studies were included for the consistent condom use outcome. Of the 15 studies included for the HIV testing uptake outcome, most (n=13, 87%) reported an improvement in HIV testing uptake, whereas 13% (2/15) of the studies reported no substantial change. Only 33% (5/15) of the studies estimated the effect size [12,13,33,34,39]; other studies (10/15, 67%) conducted chi-square tests to assess significance. For condom use behavior, most studies (10/13, 77%) reported an improvement in consistent condom use, 23% (3/13) of the studies reported no significant change, and only 23% (3/13) of the studies estimated an effect size.

The overall effect sizes estimated from the Bayesian random-effects model were 0.20 (95% CrI 0.10-0.30) for HIV testing uptake and 0.15 (95% CrI 0.05-0.26) for consistent condom use. The probability that the mean difference exceeded 0 was >99% for both HIV testing uptake and consistent condom use despite the effect sizes for a few studies (2/15, 13% for HIV testing uptake; 3/13, 23% for consistent condom use) being estimated to be close to 0. In the HIV testing uptake model, the effect sizes of 20% (3/15) of the studies seemed much larger than those of the others [33,46,48]. However, after using a robust model with *t*-distributed random effects, the pooled effect size was 0.19 (95% CrI 0.10-0.30), similar to the estimates from the original model. The probability that the mean difference exceeded 0 was still >99%. Similarly, adjusting the 15% (2/13) of the studies with large effect sizes [41,46] in the robust model for consistent condom use yielded close estimates (0.12, 95% CrI 0.05-0.23) compared to those of the original model.

**Figure 4:**
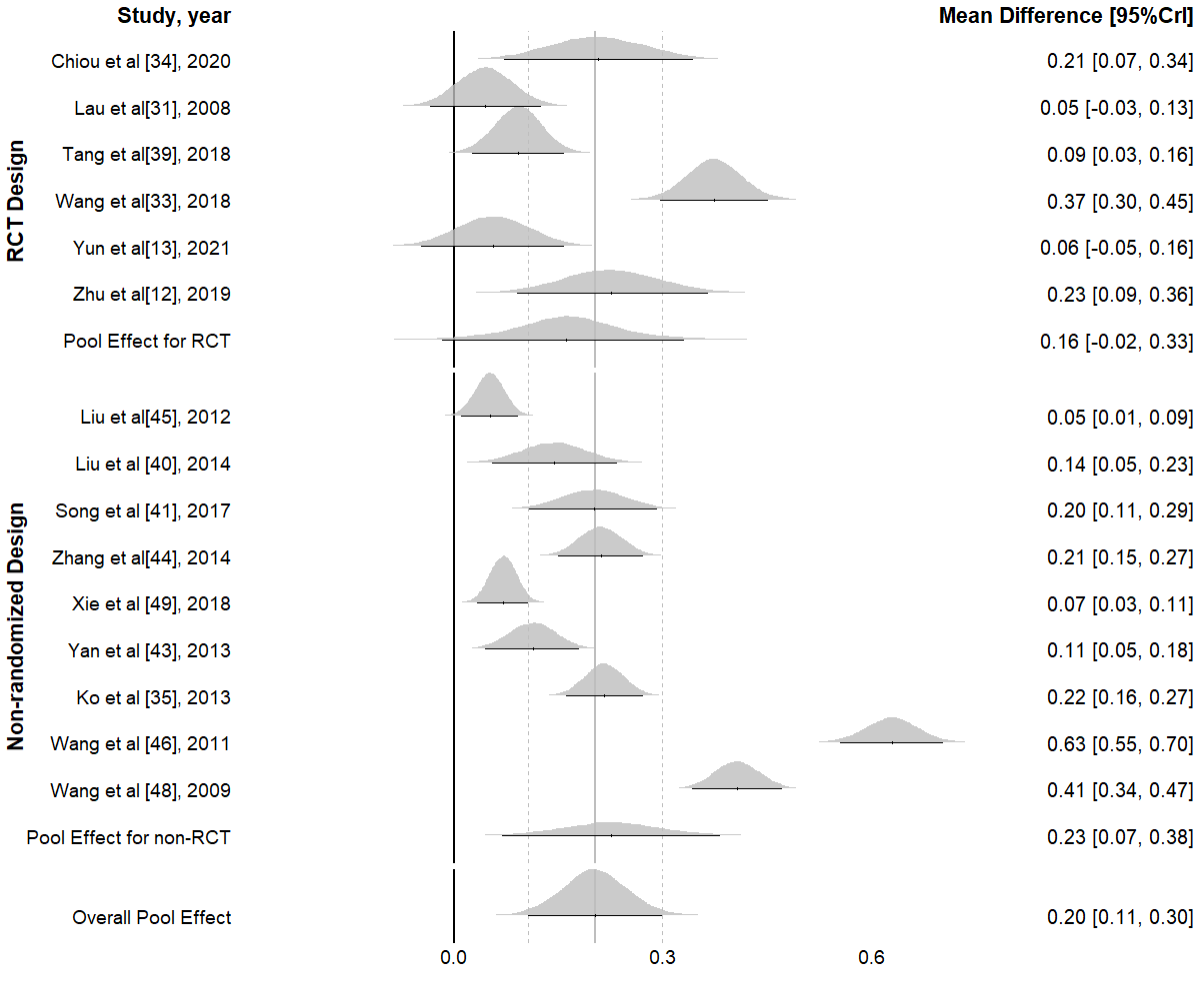
Forest plots of intervention effect on HIV testing uptake from Bayesian random-effects meta-analysis in China (2004-2021) [12,13,31,33-35,39-41,43-46,48,49]. CrI: credible interval; RCT: randomized controlled trial.

**Figure 5:**
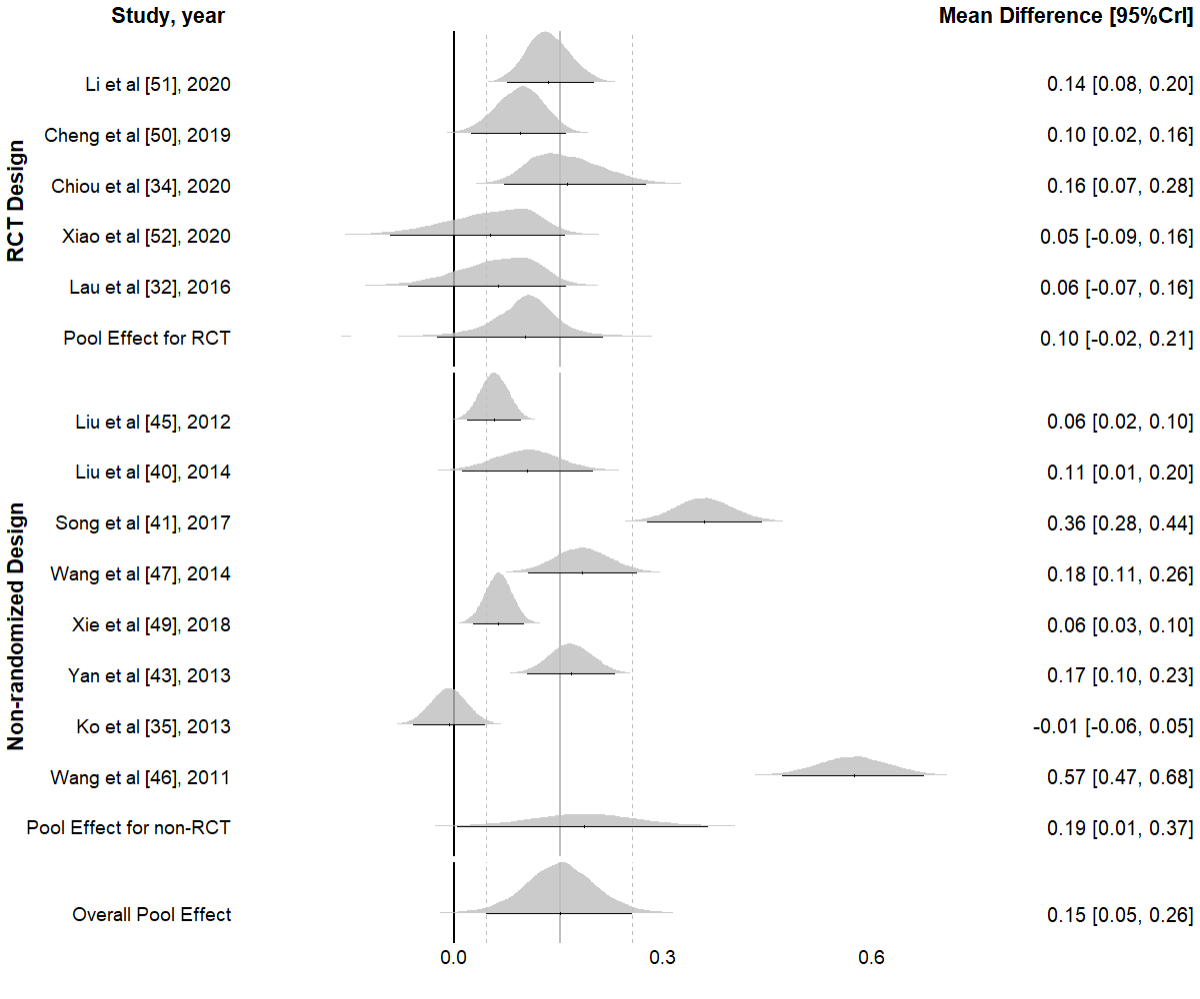
Forest plots of intervention effect on consistent condom use from Bayesian hierarchical random-effects model in China (2004-2021) [32,34,35,40,41,43,45-47,49-52]. CrI: credible interval; RCT: randomized controlled trial.

The overall effect sizes were also estimated separately by study design (RCT and nonrandomized design) given their difference in study quality assessment. Figure 4 presents the estimated effect sizes for HIV testing uptake by RCT and nonrandomized designs. The pooled effect point estimate of RCT studies (0.16, 95% CrI −0.02 to 0.33) was smaller than that of nonrandomized studies (0.23, 95% CrI 0.07-0.38), but the CrIs largely overlapped. The 95% CrI for RCTs was slightly wider than that for nonrandomized studies, but they largely overlapped. Similarly, the pooled effect point estimate for consistent condom use for RCTs (0.10, 95% CrI −0.02 to 0.21) was also smaller than that for nonrandomized studies (0.19, 95% CrI −0.00 to 0.37), but these CrIs largely overlapped (Figure 5). Detailed model results are presented in Multimedia Appendix 3.

### Covariate Analysis

We explored the association between behavior change outcomes (HIV testing uptake and consistent condom use) and study characteristics and intervention features (Table 2). Among the study characteristics and intervention features we assessed, the only characteristic that was associated with the behavior change outcome was the length of the intervention and study follow-up. Interventions lasting >6 months were associated with a 35% greater uptake of HIV testing (95% CrI 19%-51%) compared to interventions lasting 6 months. Studies followed up on for 12 months were associated with a 2% greater uptake of HIV testing (95% CrI 1%-39%) compared to those followed up on for 6 months.

**Table 2:** Association between study and intervention characteristics and study effect sizes in China (2004-2021)^a^.

Study and intervention characteristics		HIV testing uptake (n=15 studies)		Consistent condom use (n=13 studies)	
		Values	Coefficient^b^ (95% CrI^c^)	Values	Coefficient (95% CrI)
Publication year, median (IQR)		2014 (2012-2018)	−0.01 (−0.03 to 0.01)	2016 (2011-2020)	−0.01 (−0.04 to 0.02)
**Publication language, n (%)**					
	English	7 (47)	Reference	4 (31)	Reference
	Chinese	8 (53)	0.04 (−0.12 to 0.20)	9 (69)	0.10 (−0.07, 0.27)
**Outcome measure, n (%)**					
	Lifetime HIV testing	6 (40)	Reference	—^d^	—
	HIV testing in the previous 3 months	4 (27)	−0.04 (−0.24 to 0.15)	—	—
	HIV testing in the previous 6 months	5 (33)	0.09 (−0.11 to 0.27)	—	—
	Consistent condom use on the last anal sexual activity	—	—	1 (8)	Reference
	Consistent condom use in the previous month	—	—	1 (8)	−0.04 (−0.50 to 0.42)
	Consistent condom use in the previous 3 months	—	—	5 (38)	0.10 (−0.24 to 0.43)
	Consistent condom use in the previous 6 months	—	—	6 (46)	0.10 (−0.24 to 0.43)
**Intervention based on a behavior change theory, n (%)**					
	No	10 (67)	Reference	9 (69)	Reference
	Yes	5 (33)	0.05 (−0.12 to 0.21)	4 (31)	−0.10 (−0.27 to 0.07)
**Intervention included back-and-forth interactions, n (%)**					
	No	4 (27)	Reference	5 (38)	Reference
	Yes	11 (73)	0.09 (−0.08 to 0.27)	8 (62)	0.07 (−0.09 to 0.24)
**>1 intervention session, n (%)**					
	No	4 (27)	Reference	8 (62)	Reference
	Yes	11 (73)	−0.05 (−0.21 to 0.11)	5 (38)	−0.02 (−0.20 to 0.15)
**Intervention development involved the target population, n (%)**					
	No	9 (60)	Reference	8 (62)	Reference
	Yes	6 (40)	0.04 (−0.13 to 0.19)	5 (38)	−0.10 (−0.27 to 0.06)
**Intervention delivery engaged the target population, n (%)**					
	No	8 (53)	Reference	8 (62)	Reference
	Yes	7 (47)	0.04 (−0.12 to 0.21)	5 (38)	0.07 (−0.10 to 0.25)
**Length of the intervention, n (%)**					
	6 months	8 (53)	Reference	5 (38)	Reference
	<6 months	5 (33)	−0.06 (−0.17 to 0.06)	5 (38)	−0.09 (−0.27 to 0.10)
	>6 months	2 (13)	0.35 (0.19 to 0.51)	3 (23)	0.08 (−0.15 to 0.31)
**Length of study follow-up, n (%)**					
	6 months	9 (60)	Reference	7 (54)	Reference
	3 months	3 (20)	−0.14 (−0.30 to 0.02)	3 (23)	−0.10 (−0.29 to 0.09)
	12 months	3 (20)	0.02 (0.01 to 0.39)	3 (23)	0.10 (−0.12 to 0.33)

^a^Univariate regression were conducted for each characteristic.

^b^Coefficients from univariate models exploring the relationship between variables in the first column and HIV testing uptake and condom use. For example, the first coefficient represents the coefficient of publication year in a model with only publication year as the covariate.

^c^CrI: credible interval.

^d^Not applicable.

### Sensitivity Analysis

We conducted sensitivity analyses using different priors, such as a noninformative prior (μ~N[0, 10,000]) and an informative prior (μ~N[0.38, 1] for HIV testing uptake and μ~N[0.21, 1] for consistent condom use) on meta-analytic means, as well as an alternative heterogeneity prior (τ~Half-Cauchy [0,1]; Tables S1 and S2 in Multimedia Appendix 4). For both the HIV testing uptake and consistent condom use models, the effect estimates across the models were similar to those of the original models, suggesting that the estimated effects from the original models were stable.

## Discussion

### Principal Findings

Reviewing a total of 24 eligible studies published in the last 2 decades, we found promising effects for technology-based interventions designed to support HIV testing uptake and consistent condom use among men who have sex with men in China. The estimated pooled effect sizes from our primary Bayesian meta-analysis found a promising absolute effect on increasing both HIV testing and condom use—a 20% increase in HIV testing uptake and a 15% increase in consistent condom use. The probability that the effect size exceeded 0 was >99% for both HIV testing uptake and consistent condom use. To address potential outlier studies reporting larger effects compared to other reviewed studies, we used robust models for both outcomes that estimated pooled effect sizes similar to those of the original models. These promising behavior change effects of interventions in China align with findings of other meta-analyses worldwide. A previous global meta-analysis identified a significant effect in increasing HIV testing uptake (Cohen *d*=0.38) and reducing condomless anal intercourse (Cohen *d*=0.21) [20]. Another meta-analysis of computer-based interventions also estimated a significant effect in increasing condom use (Cohen *d*=0.26) [53]. To address concerns regarding prior selection, we conducted sensitivity analyses using different types of priors for both HIV testing uptake and consistent condom use. These models produced similar estimates to those of the original models. Therefore, we propose that technology-based interventions are likely to be effective in changing these 2 behaviors based on the reviewed studies.

We conducted subgroup analyses to explore the difference in estimated effect sizes across study designs to account for the varied quality of studies despite the fact that the benefit of Bayesian random-effects models is allowing for a combination of the effect sizes of studies with different designs. We found that the estimated pooled effect sizes for nonrandomized studies were slightly larger than those for RCT studies in both the HIV testing uptake and consistent condom use outcomes. The effect size dispersion of consistent condom use among RCTs seemed lower than that among nonrandomized studies. However, these 95% CrIs largely overlapped, indicating that the effect size differences between study designs were not significant or substantial. Previous meta-analyses examining the effect size difference among study designs have reported a significant estimated effect size difference in unprotected anal intercourse reduction across RCTs and nonrandomized designs, whereas the results were not significant for HIV testing uptake improvements [20,21]. These inconsistent findings suggest that study designs should be explored as an essential factor that could impact the intervention effect size in future meta-analyses. This also urges more rigorous study designs such as RCTs to fully demonstrate the effects of these technology-based interventions.

Previous reviews and meta-analyses have indicated that several intervention features might be associated with the intervention effect, including involvement of users in the design process, interactive interventions, multiple intervention sessions, longer treatment durations, and the use of combined technology modalities to deliver the intervention [20,21]. However, we did not find any significant associations between these intervention features and the effect size, which could be due to the known issue of small sample size for specific features (ie, the small number of studies included with these traits). Although our meta-regression analysis showed that interventions lasting >6 months seemed to have a significantly greater impact on HIV testing uptake compared to interventions lasting 6 months, it could be a coincidence as the 8% (2/24) of the studies that delivered interventions for >6 months reported much larger effect sizes than the rest of the studies [46,48]. Moreover, we did not find any other intervention features, such as theory-based intervention content, back-and-forth interactions, multiple intervention sessions, and community engagement, that could possibly explain the association between intervention duration and the effects on HIV testing uptake.

One of the unique features of these mobile health (mHealth) interventions in China is leveraging the same mainstream, all-in-one social networking app, WeChat. As mHealth becomes a more important and efficient tool to deliver health interventions, there are lingering questions regarding how to tailor interventions for target populations and how to scale up efficacious interventions to a broader range of populations [19,54]. Technology-based intervention modes have expanded from web-based formats to SMS text messages to social media [18,55,56]. With 1.3 billion monthly active users (>80% of the population) in China [57], WeChat is a convenient platform to distribute direct messages with text, pictures, and videos to specific users. It also offers a platform for people to develop WeChat-based apps that are less costly compared to developing a standard smartphone app [16]. In addition, using a mainstream social networking app instead of a men who have sex with men–focused app may reduce the concern of stigma by avoiding unanticipated outing or labeling. In our review, despite the fact that most studies that used WeChat only used its direct messaging function to deliver interventions, several studies (4/7, 57%) developed an official account or a mini app for risk assessment and HIV self-testing kit distribution [12,42,51,52]. Some researchers are also exploring approaches to leverage this social media platform to provide more comprehensive services together with men who have sex with men–focused community-based organizations and public opinion leaders [58-60]. Using this social networking platform, researchers may be able to enhance community engagement and provide more comprehensive health services to a larger target population.

Despite the meta-regression showing no significant association between effect size and intervention features, some patterns in intervention development and delivery can still be observed descriptively. For example, theory-based interventions have been reported to be more effective than non–theory-based ones [61]. In our review, less than a third of the studies (7/24, 29%) reported using a health behavior theory when developing the intervention. This low use of behavior change theories aligns with a previous review on mHealth studies in low- and middle-income countries [62]. This is not surprising because most of the health behavior theories were developed in the context of high-income countries, and some of the constructs, such as self-efficacy, could be difficult to apply without tailoring for the target population and local context. The insufficient use of health behavior change theories in the development of mHealth interventions highlights an opportunity to further improve the intervention effects with theory-guided intervention development and reinforces the need to develop or adapt these existing theories into culturally and contextually appropriate theories.

### Limitations

Our analysis is limited by publication bias given that significant and positive intervention results are more likely to be published. Nonsignificant findings should be encouraged to be published. All the reviewed studies (24/24, 100%) were conducted in the context of China. Despite the fact that technology-based interventions in general have been found to be effective for HIV testing and condom use behavior, the application of technology and intervention contents should be tailored based on culture, context, and the needs of the targeted populations in different countries. In addition, the data available for analysis from original study reports were limited. Future intervention studies should be encouraged to report more details about the intervention features to allow for further analysis or scale up. Moreover, we did not identify any studies that targeted transgender women in China in this review. This indicates a lack of technology-based HIV prevention research focused on transgender women in China. The lack of research on and high HIV prevalence among transgender women worldwide indicate a need for HIV prevention efforts tailored to this key population [6]. Finally, this review searched for studies published before 2021. There could be a risk of excluding relevant studies published between 2022 and 2024. These studies could be included in future reviews.

### Conclusions

Existing social networking platforms in China provide great opportunities for technology-based intervention development and distribution for HIV prevention. Technology-based HIV prevention interventions were found to have significant effects on health behavior change in both HIV testing uptake and consistent condom use among men who have sex with men in China across a broad array of studies and study designs. However, many study designs in this review were less rigorous, without a randomized design or a control group. More rigorous study designs, such as RCTs, and measurement of outcomes that address the limitations of self-report, such as picture verification of HIV self-tests, are needed to build up a more robust evidence base for the development and implementation of future technology-based intervention programs.

## Data Availability

All data generated or analyzed during this study are included in this published article (and its supplementary information files).

## Appendix

Multimedia Appendix 1 PRISMA (Preferred Reporting Items for Systematic Reviews and Meta-Analyses) checklist.

Multimedia Appendix 2 Search strategy.

Multimedia Appendix 3 Bayesian hierarchical random-effects model results.

Multimedia Appendix 4 Sensitivity analysis.

Multimedia Appendix 5 Quality assessment.

## Abbreviations

CrI: credible interval
ICROMS: integrated quality criteria for review of multiple study designs
mHealth: mobile health
PRISMA: Preferred Reporting Items for Systematic Reviews and Meta-Analyses
RCT: randomized controlled trial
