# Supplementary material for: Technology-Based HIV Prevention Interventions for Men Who Have Sex With Men: Systematic Review and Meta-Analysis
Source: J Med Internet Res. 2025 Apr 28;27:e63111. doi: 10.2196/63111 (PMC12070019; doi:10.2196/63111)
Supplement: Multimedia Appendix 2 [file jmir_v27i1e63111_app2.docx]

**Supplement 2. Search strategy**

**Search terms used for English databases**

| **Database** | **Search terms** | **Date and Results** |
| --- | --- | --- |
| PubMed | (China[All Fields] or "Hong Kong"[All Fields] or "Macau"[All Fields] or "Taiwan"[All Fields]) AND (Tongzhi[tw] or "Male"[Mesh] or male[tw] or "Homosexuality, Male"[Mesh] or "Homosexual*"[tw] or msm[tw] or "men who have sex with men"[tw] or "transgender women"[tw] or "assigned male at birth"[tw] or AMAB[tw] or "transsexual women"[tw] or "gay men"[tw] or “gay male*”[tw] or “bisexual men”[tw] or “bisexual male*”[tw]) AND ("Acquired Immunodeficiency Syndrome"[Mesh] or "Acquired Immunodeficiency Syndrome"[tw] or "HIV Infections"[Mesh] or "HIV Infections"[tw] or HIV[tw] or AIDS[tw] or "Unsafe Sex"[Mesh] or "Risk-Taking"[Mesh] or "Risk-Taking"[tw] or "Unsafe Sex"[tw]) AND ("Telemedicine"[Mesh] or "Telemedicine"[tw] or "Internet"[Mesh] or "Internet"[tw] or "Online Systems"[Mesh] or "Online Systems"[tw] or "Online Social Networking"[Mesh] "Online Social Networking"[tw] or "Smartphone"[Mesh] or "Smartphone*"[tw] or "Text Messaging"[Mesh] or "Text Messaging"[tw] or "mobile device*"[tw] or "Social Media"[Mesh] or "Social Media"[tw] or Facebook[tw] or Facetime[tw] or "Social media"[tw] or Wechat[tw] or Weibo[tw] or "web based"[tw] or online[tw] OR mHealth[tw] OR eHealth[tw] OR "mobile health"[tw] or web[tw] or "Web Browser"[Mesh] or "Mobile Applications"[Mesh] or "Mobile Applications"[tw] or app[tw] or "Cell Phone"[Mesh] or "Cell Phone"[tw]) | 10/18/2021  577 results |
| Embase | #1 'china'/exp OR 'china' OR 'hong kong'/exp OR 'hong kong' OR 'macau'/exp OR macau OR 'taiwan'/exp OR Taiwan  #2 tongzhi OR 'male'/exp OR male OR 'homosexuality, male'/exp OR 'homosexuality, male' OR 'homosexual*' OR msm OR 'men who have sex with men'/exp OR 'men who have sex with men' OR 'transgender women' OR 'assigned male at birth' OR amab OR 'transsexual women' OR 'gay men'/exp OR 'gay men' OR 'gay male*” or “bisexual men” or “bisexual male*'  #3 'acquired immunodeficiency syndrome'/exp OR 'acquired immunodeficiency syndrome' OR 'hiv infections'/exp OR 'hiv infections' OR 'hiv'/exp OR hiv OR 'aids'/exp OR aids OR 'risk-taking'/exp OR 'risk-taking' OR 'unsafe sex'/exp OR 'unsafe sex'  #4 ('telemedicine'/exp OR 'telemedicine' OR 'internet'/exp OR 'internet' OR 'online systems'/exp OR 'online systems' OR 'online social networking'/exp OR 'online social networking') AND ('online social networking'/exp OR 'online social networking') OR 'smartphone'/exp OR 'smartphone' OR 'smartphone*' OR 'text messaging'/exp OR 'text messaging' OR 'mobile device*' OR 'facebook'/exp OR facebook OR facetime OR 'social media'/exp OR 'social media' OR 'wechat'/exp OR wechat OR weibo OR 'web based' OR 'online'/exp OR online OR 'mhealth'/exp OR mhealth OR 'ehealth'/exp OR ehealth OR 'mobile health'/exp OR 'mobile health' OR 'web'/exp OR web OR 'web browser'/exp OR 'web browser' OR 'mobile applications'/exp OR 'mobile applications' OR app OR 'cell phone'/exp OR 'cell phone'  #5 #1 AND #2 AND #3 AND #4  #6 #1 AND #2 AND #3 AND #4 AND ([chinese]/lim OR [english]/lim) | 10/19/2021  622 results |
| Web of Science | TOPIC: ((Tongzhi or male or "Homosexuality, Male" or "Homosexual*" or msm or "men who have sex with men" or "transgender women" or "assigned male at birth" or AMAB or "transsexual women" or "gay men" or “gay male*” or “bisexual men” or “bisexual male*”)) AND TOPIC: (("Acquired Immunodeficiency Syndrome" or "HIV Infections" or HIV or AIDS or "Unsafe Sex" or "Risk-Taking" or "Unsafe Sex")) AND TOPIC: (("Telemedicine" or "Internet" or "Online Systems" or "Online Social Networking" "Online Social Networking" or "Smartphone" or "Smartphone*" or "Text Messaging" or "Text Messaging" or "mobile device*" or "Social Media" or "Social Media" or Facebook or Facetime or "Social media" or Wechat or Weibo or "web based" or online OR mHealth OR eHealth OR "mobile health" or web or "Web Browser" or "Mobile Applications" or "Mobile Applications" or app or "Cell Phone")) AND PUBLICATION YEARS: ( 2021 OR 2016 OR 2011 OR 2007 OR 2020 OR 2015 OR 2010 OR 2006 OR 2019 OR 2014 OR 2009 OR 2005 OR 2018 OR 2013 OR 2008 OR 2017 OR 2012 ) AND LANGUAGES: ( ENGLISH )  Timespan: All years. Indexes: SCI-EXPANDED, SSCI, A&HCI, CPCI-S, CPCI-SSH, BKCI-S, BKCI-SSH, ESCI, CCR-EXPANDED, IC. | 10/19/2021  334 results |

**Search terms used for Chinese databases**

"中国" or "香港" or "澳门" or "台湾" AND "男男" or "MSM" or "同性恋" or "同志" or "男男性行为" or "双性恋" AND "艾滋病" or "AIDS" or "HIV" AND "预防" or "干预" or "intervention" AND "新媒体" or "短信干预" or "远程会议" or "微信" or "网络" or "eHealth" or "应用程序" or "远距离医学" or "手机" or "应用" or "社交媒体" or "社交网络" or "微博"
