# Supplementary material for: Technology-Based HIV Prevention Interventions for Men Who Have Sex With Men: Systematic Review and Meta-Analysis
Source: J Med Internet Res. 2025 Apr 28;27:e63111. doi: 10.2196/63111 (PMC12070019; doi:10.2196/63111)
Supplement: Multimedia Appendix 3 [file jmir_v27i1e63111_app3.docx]

**Supplement 3. Bayesian hierarchical random effects model results.**

**Model:**

**Observation level model**

$$\boldsymbol{y}_{\boldsymbol{j}}\boldsymbol{|}\boldsymbol{\theta}_{\boldsymbol{j}}\boldsymbol{,}\boldsymbol{\sigma}_{\boldsymbol{j}}\boldsymbol{\sim N(}\boldsymbol{\theta}_{\boldsymbol{j}}\boldsymbol{,}\boldsymbol{\sigma}_{\boldsymbol{j}}^{\boldsymbol{2}}\boldsymbol{)}$$

**Study effects model**

$$\boldsymbol{\theta}_{\boldsymbol{j}}\boldsymbol{|\mu,\tau\sim}\boldsymbol{N}\left( \boldsymbol{\mu,}\boldsymbol{\tau}^{\boldsymbol{2}} \right)$$

$$\boldsymbol{\mu\sim N}\left( \boldsymbol{0,1} \right)$$

$$\boldsymbol{\tau\sim}\boldsymbol{t}_{\boldsymbol{3}}\boldsymbol{(0,1)}$$

**Study effects model (with robust random effects distribution)**

$$\boldsymbol{\theta}_{\boldsymbol{j}}\boldsymbol{|\mu,\tau\sim}\boldsymbol{t}_{\boldsymbol{\nu}}\left( \boldsymbol{\mu,}\boldsymbol{\tau}^{\boldsymbol{2}} \right)$$

$$\boldsymbol{\mu\sim N}\left( \boldsymbol{0,1} \right)$$

$$\boldsymbol{\tau\sim}\boldsymbol{t}_{\boldsymbol{3}}\left( \boldsymbol{0,1} \right)$$

$$\boldsymbol{\nu\sim G(2, 0.1)}$$

**Estimation:**

**HMC using four chains run for 20,000 iterations with 10,000 removed as burn-in. We conducted standard diagnostics for absence of convergence and checked for divergent transitions and found no obvious issues.**

**Table S1. Bayesian hierarchical random effects model results.**

| Parameter | Posterior mean | SD | Lower CrL | Upper CrL |
| --- | --- | --- | --- | --- |
| **Model for HIV testing uptake (both RCT and non-randomized studies)** | | | | |
| $\mu$ | 0.20 | 0.05 | 0.12 | 0.28 |
| $\boldsymbol{\tau}$ | 0.18 | 0.04 | 0.13 | 0.26 |
| Liu et al., 2012 | -0.15 | 0.05 | -0.24 | -0.07 |
| Liu et al., 2014 | -0.06 | 0.07 | -0.17 | 0.05 |
| Song et al., 2017 | 0 | 0.07 | -0.11 | 0.10 |
| Zhang et al., 2014 | 0.01 | 0.06 | -0.09 | 0.10 |
| Chiou et al., 2020 | 0.01 | 0.08 | -0.13 | 0.15 |
| Xie et al., 2018 | -0.13 | 0.05 | -0.22 | -0.05 |
| Yan et al., 2013 | -0.09 | 0.06 | -0.19 | 0.01 |
| Ko et al., 2013 | 0.01 | 0.06 | -0.08 | 0.10 |
| Lau et al., 2008 | -0.16 | 0.06 | -0.26 | -0.06 |
| Wang et al., 2011 | 0.42 | 0.06 | 0.32 | 0.52 |
| Tang et al., 2018 | -0.11 | 0.06 | -0.21 | -0.01 |
| Wang et al., 2018 | 0.17 | 0.06 | 0.08 | 0.27 |
| Yun et al., 2021 | -0.15 | 0.07 | -0.26 | -0.03 |
| Zhu et al., 2019 | 0.03 | 0.08 | -0.10 | 0.16 |
| Wang et al., 2009 | 0.20 | 0.06 | 0.11 | 0.30 |
| **Model for HIV testing uptake (RCT only)** | | | | |
| $\mu$ | 0.16 | 0.09 | 0.03 | 0.30 |
| $\boldsymbol{\tau}$ | 0.19 | 0.10 | 0.09 | 0.36 |
| Chiou et al., 2020 | 0.05 | 0.10 | -0.11 | 0.21 |
| Lau et al., 2008 | -0.12 | 0.09 | -0.27 | 0.03 |
| Tang et al., 2018 | -0.07 | 0.09 | -0.21 | 0.08 |
| Wang et al., 2018 | 0.21 | 0.09 | 0.07 | 0.36 |
| Yun et al., 2021 | -0.10 | 0.10 | -0.26 | 0.04 |
| Zhu et al., 2019 | 0.06 | 0.10 | -0.10 | 0.24 |
| **Model for HIV testing uptake (non-randomized studies only)** | | | | |
| $\mu$ | 0.23 | 0.08 | 0.10 | 0.35 |
| $\boldsymbol{\tau}$ | 0.22 | 0.07 | 0.14 | 0.36 |
| Liu et al., 2012 | -0.17 | 0.08 | -0.30 | -0.05 |
| Liu et al., 2014 | -0.08 | 0.09 | -0.22 | 0.06 |
| Song et al., 2017 | -0.03 | 0.09 | -0.17 | 0.12 |
| Zhang et al., 2014 | -0.02 | 0.08 | -0.15 | 0.12 |
| Xie et al., 2018 | -0.15 | 0.08 | -0.28 | -0.03 |
| Yan et al., 2013 | -0.11 | 0.08 | -0.25 | 0.02 |
| Ko et al., 2013 | -0.01 | 0.08 | -0.14 | 0.12 |
| Wang et al., 2011 | 0.40 | 0.08 | 0.27 | 0.54 |
| Wang et al., 2009 | 0.18 | 0.08 | 0.05 | 0.32 |
| **Model for consistent condom use (both RCT and non-randomized studies)** | | | | |
| $\mu$ | 0.15 | 0.05 | 0.07 | 0.24 |
| $\boldsymbol{\tau}$ | 0.18 | 0.05 | 0.12 | 0.26 |
| Liu et al., 2012 | -0.09 | 0.06 | -0.18 | 0 |
| Liu et al., 2014 | -0.05 | 0.07 | -0.16 | 0.06 |
| Song et al., 2017 | 0.20 | 0.06 | 0.10 | 0.31 |
| Li et al., 2020 | -0.01 | 0.06 | -0.10 | 0.09 |
| Cheng et al., 2019 | -0.06 | 0.06 | -0.16 | 0.04 |
| Chiou et al., 2020 | 0.05 | 0.07 | -0.07 | 0.17 |
| Wang et al., 2014 | 0.03 | 0.06 | -0.07 | 0.13 |
| Xiao et al., 2020 | -0.13 | 0.08 | -0.27 | 0 |
| Xie et al., 2018 | -0.09 | 0.06 | -0.18 | 0 |
| Yan et al., 2013 | 0.01 | 0.06 | -0.08 | 0.11 |
| Ko et al., 2013 | -0.16 | 0.06 | -0.25 | -0.07 |
| Lau et al., 2016 | -0.11 | 0.08 | -0.24 | 0.02 |
| Wang et al., 2011 | 0.41 | 0.07 | 0.29 | 0.53 |
| **Model for consistent condom use (RCT only)** | | | | |
| $\mu$ | 0.10 | 0.06 | 0.01 | 0.18 |
| $\boldsymbol{\tau}$ | 0.09 | 0.07 | 0.01 | 0.23 |
| Li et al., 2020 | 0.03 | 0.06 | -0.05 | 0.14 |
| Cheng et al., 2019 | -0.01 | 0.06 | -0.10 | 0.09 |
| Chiou et al., 2020 | 0.06 | 0.07 | -0.03 | 0.19 |
| Xiao et al., 2020 | -0.05 | 0.07 | -0.18 | 0.04 |
| Lau et al., 2016 | -0.04 | 0.07 | -0.17 | 0.05 |
| **Model for consistent condom use (non-randomized studies only)** | | | | |
| $\mu$ | 0.19 | 0.09 | 0.04 | 0.33 |
| $\boldsymbol{\tau}$ | 0.24 | 0.09 | 0.14 | 0.41 |
| Liu et al., 2012 | -0.13 | 0.09 | -0.28 | 0.02 |
| Liu et al., 2014 | -0.08 | 0.10 | -0.24 | 0.08 |
| Song et al., 2017 | 0.17 | 0.10 | 0.02 | 0.33 |
| Wang et al., 2014 | 0 | 0.10 | -0.16 | 0.16 |
| Xie et al., 2018 | -0.12 | 0.09 | -0.27 | 0.03 |
| Yan et al., 2013 | -0.02 | 0.10 | -0.17 | 0.14 |
| Ko et al., 2013 | -0.19 | 0.09 | -0.35 | -0.04 |
| Wang et al., 2011 | 0.39 | 0.10 | 0.23 | 0.56 |
| **Models for HIV testing uptake, controlling for study quality** | | | | |
| $\mu$ | 0.21 | 0.05 | 0.13 | 0.30 |
| $\boldsymbol{\tau}$ | 0.16 | 0.04 | 0.10 | 0.24 |
| Good quality^1^ | -0.14 | 0.14 | -0.37 | 0.07 |
| Liu et al., 2012 | -0.16 | 0.06 | -0.25 | -0.07 |
| Liu et al., 2014 | -0.07 | 0.07 | -0.18 | 0.04 |
| Song et al., 2017 | -0.01 | 0.07 | -0.12 | 0.10 |
| Zhang et al., 2014 | 0 | 0.06 | -0.10 | 0.09 |
| Chiou et al., 2020 | 0 | 0.08 | -0.13 | 0.14 |
| Xie et al., 2018 | -0.14 | 0.05 | -0.23 | -0.05 |
| Yan et al., 2013 | -0.10 | 0.06 | -0.20 | 0 |
| Ko et al., 2013 | 0 | 0.06 | -0.09 | 0.10 |
| Lau et al., 2008 | -0.17 | 0.06 | -0.27 | -0.06 |
| Wang et al., 2011 | 0.41 | 0.06 | 0.31 | 0.52 |
| Tang et al., 2018 | 0.02 | 0.12 | -0.18 | 0.22 |
| Wang et al., 2018 | 0.16 | 0.06 | 0.06 | 0.27 |
| Yun et al., 2021 | -0.02 | 0.12 | -0.23 | 0.18 |
| Zhu et al., 2019 | 0.02 | 0.08 | -0.11 | 0.15 |
| Wang et al., 2009 | 0.19 | 0.06 | 0.09 | 0.29 |
| **Models for consistent condom use, controlling for study quality** | | | | |
| $\mu$ | 0.16 | 0.06 | 0.07 | 0.25 |
| $\boldsymbol{\tau}$ | 0.16 | 0.05 | 0.09 | 0.25 |
| Good quality^1^ | -0.10 | 0.14 | -0.33 | 0.13 |
| Liu et al., 2012 | -0.10 | 0.06 | -0.20 | 0 |
| Liu et al., 2014 | -0.05 | 0.07 | -0.16 | 0.06 |
| Song et al., 2017 | 0.20 | 0.07 | 0.09 | 0.31 |
| Li et al., 2020 | -0.01 | 0.06 | -0.11 | 0.09 |
| Cheng et al., 2019 | 0.03 | 0.13 | -0.18 | 0.24 |
| Chiou et al., 2020 | 0.04 | 0.07 | -0.08 | 0.16 |
| Wang et al., 2014 | 0.03 | 0.07 | -0.08 | 0.13 |
| Xiao et al., 2020 | -0.13 | 0.09 | -0.28 | 0.01 |
| Xie et al., 2018 | -0.09 | 0.06 | -0.19 | 0 |
| Yan et al., 2013 | 0.01 | 0.06 | -0.09 | 0.11 |
| Ko et al., 2013 | -0.16 | 0.06 | -0.26 | -0.06 |
| Lau et al., 2016 | -0.03 | 0.13 | -0.24 | 0.18 |
| Wang et al., 2011 | 0.41 | 0.08 | 0.29 | 0.54 |

^1^ The quality of study was assessed by integrated quality criteria for the review of multiple study designs (ICROM) [27] and coded into a dummy variable (0 indicates that the study does not meet either the mandatory criteria or minimum score; 1 indicates that both mandatory criteria and minimum score are met).

Reference

27. Zingg W, Castro-Sanchez E, Secci FV, Edwards R, Drumright LN, Sevdalis N, et al. Innovative tools for quality assessment: integrated quality criteria for review of multiple study designs (ICROMS). Public Health. 2016 Apr;133:19-37. PMID: 26704633. doi: 10.1016/j.puhe.2015.10.012.
