# Supplementary material for: Technology-Based HIV Prevention Interventions for Men Who Have Sex With Men: Systematic Review and Meta-Analysis
Source: J Med Internet Res. 2025 Apr 28;27:e63111. doi: 10.2196/63111 (PMC12070019; doi:10.2196/63111)
Supplement: Multimedia Appendix 4 [file jmir_v27i1e63111_app4.docx]

**Supplement 4. Sensitivity analysis.**

**Table S1. Model estimates for sensitivity analysis for HIV testing uptake.**

|  | Posterior Mean | SD | Lower CrL | Upper CrL | Original Posterior Mean | % Deviation |
| --- | --- | --- | --- | --- | --- | --- |
| **Non-informative prior** **on meta analytic mean:** $\mu$ **prior ~ N(0, 10000)** | | | | | | |
| $\mu$ | 0.21 | 0.05 | 0.12 | 0.28 | 0.19 | 8.08 |
| $\boldsymbol{\tau}$ | 0.18 | 0.04 | 0.13 | 0.26 | 0.16 | 11.70 |
| **Informative priors on meta analytic mean:** $\mu$ **prior ~ N(0.38, 1)** | | | | | | |
| $\mu$ | 0.20 | 0.05 | 0.13 | 0.28 | 0.19 | 7.49 |
| $\boldsymbol{\tau}$ | 0.18 | 0.04 | 0.13 | 0.25 | 0.16 | 10.37 |
| **Alternative heterogeneity prior:** $\boldsymbol{\tau}$ **prior ~ HC(0, 1)** | | | | | | |
| $\mu$ | 0.20 | 0.05 | 0.12 | 0.28 | 0.19 | -74.10 |
| $\boldsymbol{\tau}$ | 0.18 | 0.04 | 0.13 | 0.26 | 0.16 | -75.23 |

Note: The intercept prior N(0.38, 1) was based on findings from a previous meta-analysis study [20]. The “original posterior mean” column presents estimates from the robust model (Table 2). CrL = 95% credible interval; ESS = Effective sample size; % Deviation=(Mean – Original Mean)/ Original Mean*100; Tau represents the between-study heterogeneity.

**Table S2. Model estimates for sensitivity analysis for consistent condom use**

|  | Posterior Mean | SD | Lower CrL | Upper CrL | Original Posterior Mean | % Deviation |
| --- | --- | --- | --- | --- | --- | --- |
| **Non-informative priors on meta analytic mean:** $\mu$ **prior ~ N(0, 10000)** | | | | | | |
| $\mu$ | 0.15 | 0.05 | 0.07 | 0.24 | 0.14 | 11.10 |
| $\boldsymbol{\tau}$ | 0.18 | 0.05 | 0.12 | 0.26 | 0.15 | 18.18 |
| **Informative priors on meta analytic mean:** $\mu$ **prior ~ N(0.21, 1)** | | | | | | |
| $\mu$ | 0.15 | 0.05 | 0.07 | 0.24 | 0.14 | 10.93 |
| $\boldsymbol{\tau}$ | 0.18 | 0.04 | 0.12 | 0.26 | 0.15 | 16.22 |
| **Alternative heterogeneity prior:** $\boldsymbol{\tau}$ **prior ~ HC(0, 1)** | | | | | | |
| $\mu$ | 0.15 | 0.05 | 0.06 | 0.24 | 0.14 | 10.45 |
| $\boldsymbol{\tau}$ | 0.18 | 0.05 | 0.12 | 0.27 | 0.15 | 17.8 |

Note: The intercept prior N(0.38, 1) was based on findings from a previous meta-analysis study [20]. The “original posterior mean” column presents estimates from the robust model (Table 2). CrL = 95% credible interval; ESS = Effective sample size; % Deviation=(Mean – Original Mean)/ Original Mean*100; Tau represents the between-study heterogeneity.

Reference

20. Xin M, Viswanath K, Li AY, Cao W, Hu Y, Lau JT, et al. The Effectiveness of Electronic Health Interventions for Promoting HIV-Preventive Behaviors Among Men Who Have Sex With Men: Meta-Analysis Based on an Integrative Framework of Design and Implementation Features. J Med Internet Res. 2020 May 25;22(5):e15977. PMID: 32449685. doi: 10.2196/15977.
