# Supplementary material for: Technology-Based HIV Prevention Interventions for Men Who Have Sex With Men: Systematic Review and Meta-Analysis
Source: J Med Internet Res. 2025 Apr 28;27:e63111. doi: 10.2196/63111 (PMC12070019; doi:10.2196/63111)
Supplement: Multimedia Appendix 5 [file jmir_v27i1e63111_app5.docx]

**Supplement 5. Quality assessment**

**Table S1. Study quality assessment using ICROMS.**

| Author, year | Study design | Global quality score | Minimum  score met | Mandatory criteria met |
| --- | --- | --- | --- | --- |
| Li et al., 2020 | RCT | 24 | Yes | No |
| Cheng et al., 2019 | RCT | 28 | Yes | Yes |
| Chiou et al., 2020 | RCT | 27 | Yes | No |
| Xiao et al., 2020 | RCT | 27 | Yes | No |
| Lau et al., 2008 | RCT | 29 | Yes | No |
| Lau et al., 2016 | RCT | 30 | Yes | Yes |
| Luo et al., 2021 | RCT | 19 | No | No |
| Tang et al., 2016 | RCT | 31 | Yes | Yes |
| Tang et al., 2018 | RCT | 31 | Yes | Yes |
| Wang et al., 2018 | RCT | 29 | Yes | No |
| Yun et al., 2021 | RCT | 31 | Yes | Yes |
| Zhu et al., 2019 | RCT | 31 | Yes | No |
| Tang et al., 2019 | RCT | 30 | Yes | Yes |
| Ko et al., 2013 | CBA | 16 | No | No |
| Liu et al., 2012 | NCBA | 20 | No | No |
| Liu et al., 2014 | NCBA | 21 | No | No |
| Song et al., 2017 | NCBA | 25 | Yes | No |
| Zhang et al., 2014 | NCBA | 22 | Yes | No |
| Wang et al., 2014 | NCBA | 16 | No | No |
| Xie et al., 2018 | NCBA | 23 | Yes | No |
| Yan et al., 2013 | NCBA | 22 | Yes | No |
| Tao et al., 2020 | NCBA | 21 | No | No |
| Wang et al., 2011 | NCBA | 22 | Yes | No |
| Wang et al., 2009 | NCBA | 19 | No | No |

* The minimum required global quality scores are 22 for both randomized controlled trial (RCT) and non-controlled before-after (NCBA) studies, and 18 for controlled before-after (CBA) study based on integrated quality criteria for review of multiple study designs (ICROMS).
